# Mineral Composition Analysis of Red Horse-Chestnut (*Aesculus × Carnea*) Seeds and Hydroalcoholic Crude Extract Using ICP OES

**DOI:** 10.3390/molecules30040819

**Published:** 2025-02-10

**Authors:** Veronica D’Eusanio, Andrea Marchetti, Mirco Rivi, Lorenzo Morelli, Paolina Scarponi, Luca Forti, Lorenzo Tassi

**Affiliations:** 1 Department of Chemical and Geological Sciences, University of Modena and Reggio Emilia, 41125 Modena, Italy; andrea.marchetti@unimore.it (A.M.); mirco.rivi@unimore.it (M.R.); lorenzo.morelli@unimore.it (L.M.); paolina.scarponi@unimore.it (P.S.); luca.forti@unimore.it (L.F.); 2 National Interuniversity Consortium of Materials Science and Technology (INSTM), 50121 Firenze, Italy; 3 Interdepartmental Research Center BIOGEST-SITEIA, University of Modena and Reggio Emilia, 42124 Reggio Emilia, Italy; 4 Department of Life Sciences, University of Modena and Reggio Emilia, 41125 Modena, Italy

**Keywords:** red horse-chestnut, *Aesculus × carnea* seeds, metal fraction, ICP OES

## Abstract

This study presents findings on the metal and metalloid composition of red horse-chestnut (*Aesculus* × *carnea*, *AXC*) seeds, determined by the ICP OES technique. Samples were collected from five AXC plants located in Modena (Italy) over four consecutive years (2016–2019). The seeds underwent proximate analysis, which included measurements of moisture content, proteins, lipids, carbohydrates, ash, and elemental composition. The analysis revealed consistent values for these parameters throughout the study period. The metal content of the AXC seeds can be categorized into two groups: (i) major components, with concentrations ranging from 1 to <1500 mg/100 g dry basis (d.b.), where K was identified as the most abundant element, and (ii) minor constituents, with concentrations between 1 and <1000 μg/100 g d.b., with Li, Mo, and Ti at the lower concentration limit. Comparative analyses were performed using literature data on AHP and AHH seeds, which, like AXC, belong to the *Sapindaceae* family and were collected from the same area and period. A hydroalcoholic extract of AXC seeds was prepared annually and characterized, with results compared to a commercial product (AXC_herb). AXC extracts had approximately 30% higher analyte concentrations than AXC_herb, while AXC seeds showed 20–30% higher metal and metalloid levels than AHP and AHH.

## 1. Introduction

Traditional medicine has long relied on empirical observations, using natural sources to develop remedies for a wide range of ailments. Over centuries, these practices have provided the foundation for modern pharmacology, as many bioactive compounds initially discovered in traditional medicine have been scientifically validated for their therapeutic effects [1,2,3]. With advancements in analytical techniques and a deeper understanding of pharmacological mechanisms, research has increasingly focused on identifying, isolating, and characterizing active compounds from medicinal plants. This integration of traditional knowledge with scientific methodologies has led to the formal recognition of numerous plant-derived substances, particularly those requiring minimal processing, in modern pharmacopeias [4,5]. As a result, interest in naturally occurring bioactive compounds has expanded beyond conventionally recognized medicinal plants to include a broader range of species, including those primarily cultivated for ornamental purposes.

Ornamental plants from the *Sapindaceae* family, particularly those in the *Hippocastanoideae* subfamily, are of significant interest due to the bioactive components of their seeds. These species, which are widely cultivated as avenue trees and ornamental gardens across Europe, have been identified as sources of pharmacologically active molecules with potential therapeutic applications. However, within this family, *Aesculus × carnea* (AXC), commonly known as red horse-chestnut, has been relatively underexplored compared to other species such as *Aesculus hippocastanum* pure species (AHP) and its hybrid (AHH). Previous studies have largely concentrated on AHP and AHH, which are distinguished by their floral coloration and slight morphological variation [6,7,8,9]. In contrast, AXC is notable for its striking red spring flowers, although other distinguishing characteristics require expert botanical knowledge.

The existing literature suggests that although the seeds of these plants share morphological and chemical similarities, their specific pharmacological profiles may differ. AHP and AHH have been studied for their anti-inflammatory, antioxidant, and venotonic properties, primarily due to their high escin and flavonoid content. AXC, despite being a hybrid of these species, has received limited scientific attention. However, its seeds contain higher levels of proanthocyanidins and tannins, which may contribute to enhanced antioxidant and antimicrobial activities [7]. For this reason, AXC is recognized for its pharmacological and health-promoting properties, which are attributed to bioactive compounds found in its seeds and other plant tissues. Among these, escin—a mixture of saponins—stands out, alongside the glucoside esculin. AXC exhibits anti-inflammatory [8], antioxidant [9], analgesic, antimicrobial [10], vasoprotective, venotonic, and anti-edematous effects [5,11]. Floral extracts of AXC, rich in flavonoids, flavanols, free phenolic acids, and coumarins, demonstrate enhanced radical scavenging activity against reactive oxygen species (ROS), particularly hydroxyl and peroxyl radicals, due to synergistic interactions among these compounds [10,11]. Recent studies have confirmed that these phytotherapeutic preparations offer strong protection to plasma biomolecules, significantly limiting oxidative and nitrative damage induced by peroxynitrite [12]. This mechanism inhibits both protein nitration and lipid peroxidation, leading to a marked reduction in 3-nitrotyrosine levels and thiobarbituric acid-reactive molecules. Furthermore, the high total polyphenol content (TPC) in AXC extracts prevents plasma thiol depletion and enhances the non-enzymatic antioxidant capacity of blood plasma. In addition to these mechanisms, AXC extracts exhibit mild anti-aggregant and anticoagulant properties, which may contribute to their therapeutic benefits in venous circulatory disorders. Importantly, these properties allow for safe use in multi-therapeutic regimens alongside anticoagulant medications, minimizing the risk of adverse interactions.

The existing literature on AXC is limited and primarily focuses on its bioactive components. However, little attention has been given to the elemental composition of AXC seeds, despite the crucial role that metals and metalloids play in plant metabolism and therapeutic potential. The gap in research regarding the elemental composition and bioactive potential of AXC seeds represents a critical area of investigation. Studying the elemental composition of AXC seeds is crucial for multiple reasons. Metals and metalloids play a fundamental role in plant physiology, influencing biochemical pathways, enzymatic activities, and secondary metabolite production. In medicinal plants, trace elements such as iron (Fe), zinc (Zn), and copper (Cu) can enhance pharmacological properties, while others, like cadmium (Cd) and lead (Pb), may pose toxicological risks. Despite the recognized bioactive potential of *Aesculaceae* species, limited data are available on the elemental composition of AXC seeds, particularly in relation to their pharmacological and nutraceutical applications. Quantifying essential and potentially harmful elements in AXC extracts is necessary to assess their safety for therapeutic use and to ensure compliance with pharmacopeial standards. A comparative analysis between AXC, AHP, and AHH provides critical insights into the uptake and accumulation of elements in different *Aesculus* species. This comparison allows for an assessment of species-specific metal uptake mechanisms. Since all three cultivars were collected from the same geographical area under identical soil and climatic conditions, differences in their elemental composition could indicate species-dependent variations in metal absorption, translocation, and accumulation. Such findings could have broader implications for understanding how different *Aesculus* species interact with their environment, which is particularly relevant for their potential cultivation and use in phytotherapy. This study, therefore, contributes not only to the pharmacological evaluation of AXC but also to a deeper understanding of how genetic factors influence elemental uptake in medicinal plants.

This investigation aimed to identify potential contaminants as well as essential macroelements, which not only enhance the quality of the extract but also contribute significantly to its value and broader applications. To address this, this study focused on analyzing the elemental composition of metals and metalloids in AXC seeds and their medicinal extracts, prepared according to pharmacopeial guidelines. By examining samples collected over four consecutive years (2016–2019), the research seeks to establish potential correlations between seed composition and pharmacological properties. The methodology follows established protocols from previous studies on AHP and AHH seeds, allowing for a comparative analysis of these species within the *Sapindaceae* family [6,7,8,9].

## 2. Results and Discussion

Immediately after seed collection, biometric and morphological parameters were measured, including the total seed mass, along with the minimum and maximum values related to a set of at least 100 seeds, the pericarp fraction (woody bark + episperm) as a percentage, the kernel fraction as a percentage, and the seed shape. The results are presented in Table 1. Mass was measured using a precision balance (Mettler MX2002/M, Mettler Toledo, Columbus, OH, USA) with a sensitivity of ±0.01 g.

Analysis of the data shows that, in 2017, fruiting produced the smallest seeds on average, whereas 2016 produced the largest seeds. Consequently, these morphometric characteristics influenced all parameters listed in Table 1.

### 2.1. Proximate Analysis of Seeds

Proximate analysis serves as a fundamental initial step in the characterization of plant matrices [2,3]. Table 2 presents the proximate composition of AXC over four consecutive years of investigation. The table summarizes the experimental data for the main components of *Aesculus* seeds, along with relevant parameters related to soluble fractions.

The statistical analysis indicates that cultivar–year interactions had no significant effect on the proximate composition of the seeds (*p* > 0.05), suggesting that the nutritional profile of AXC remains stable across different harvest years. The variability in composition across years was minimal, with only slight fluctuations observed. The humidity percentage in fresh seeds ranged from 50.2% (2017) to 50.7% (2018), averaging 50.4% across all four years. Similarly, residual moisture content after natural drying remained consistent, with values between 10.0% and 10.5%. The protein content ranged from 3.11% to 3.37%, while lipid levels fluctuated slightly between 4.27% and 4.56%. Carbohydrate content remained stable, with values between 15.2% and 15.8%, and ash content showed no significant variation, indicating a well-conserved mineral composition. Cold water solubility (CWS) and total inorganic soluble salts (TISS) were also assessed as indicators of the physicochemical properties of AXC seeds. The CWS values, ranging from 54.5% to 55.1%, suggest a high proportion of hydrophilic organic molecules, likely due to the presence of soluble carbohydrates and proteins. The TISS values, fluctuating between 2.69% and 2.81%, are consistent with the ash content, reflecting the mineral fraction’s contribution to solubility.

The elemental composition of AXC seeds reveals some interesting characteristics. The average carbon (C%) content in AXC seeds was approximately 1% higher than reported values for other *Hippocastanaceae* seeds, with similar trends observed for nitrogen (N%) and hydrogen (%) content [13]. Interestingly, sulfur (S%) was not detectable in AXC seeds, in contrast to *Hippocastanaceae* seeds, where it is typically present. The absence of free sulfur suggests that it may exist primarily in bound forms, such as in sulfur-containing amino acids like cysteine or methionine, but at concentrations below the detection limit (<0.1%) of the elemental analysis system.

Table 3 summarizes the concentrations of various elements in mineralized AXC seed flour samples.

A thorough examination of Table 3 reveals two distinct groups of elements: (i) major elements and (ii) minor elements.

(i) Major elements: This group comprised elements present in significant quantities, with concentrations ranging from 1 to 1500 mg/100 g (dry basis). The elements can be ordered by their maximum observed concentrations as follows:

K (1322) > P (871) > Mg (100) > Ca (59.9) > Na (9.39) > Fe (4.65) > Al (3.27) > Cu (1.95) > Zn (1.90).

Values in parentheses represent the maximum concentrations observed during the study period.

Among these, K was the most abundant element across all the samples, accounting for approximately 50% of the ash content. Similar findings have been reported for seeds of other *Hippocastanaceae* species, such as AHP and AHH, with data for these species taken from the relevant literature [9].

Figures S1–S6 illustrate the comparative concentrations of selected major elements in AXC seeds, which are the primary focus of this study, along with reference data for AHP and AHH [9]. These comparisons help contextualize the elemental profile of AXC within the broader *Hippocastanaceae* family.

In Figure S2, the K concentration in AXC is consistently approximately 10% higher than that in AHP and approximately 30–35% higher than that in AHH. Interestingly, the AHH species, a pink-flowered horse chestnut, has been hypothesized to be a hybrid of AHP and AXC. Surprisingly, AXC showed a greater deviation from AHH in K content compared to its divergence from the pure AHP species. A similar distribution pattern was observed for Zn, albeit at concentrations 2–3 orders of magnitude lower than those of K (Figure S3). Zn and K are essential elements for plant metabolism, playing crucial roles in physiological and biochemical processes [14,15,16,17]. The higher Zn and K levels in AXC compared to AHP and AHH probably indicate possible species-specific accumulation patterns that warrant further investigation. Zn uptake in plants is influenced by soil composition, pH, and interactions with other metals, particularly Fe and Cu [18,19]. The relative stability of Fe concentrations in AXC seeds despite high Zn levels suggests effective homeostatic regulation, preventing competition for absorption sites. Environmentally, Zn is introduced into soils through natural weathering, fertilizers, and industrial pollution. While excessive Zn can be toxic to plants, leading to growth inhibition and oxidative damage, the concentrations observed in AXC seeds fall within typical ranges for edible nuts and seeds, indicating no significant phytotoxicity risk [20]. On the other hand, the high K content in AXC seeds may have implications for both plant physiology and medicinal applications. In plants, K enhances nutrient translocation and improves stress tolerance, while in humans, dietary K intake is associated with cardiovascular health benefits, including blood pressure regulation [16,17]. The consistent presence of high K levels in AXC seeds reinforces their potential value in phytotherapy, particularly in formulations targeting vascular health [21].

For Cu, the AXC species exhibited intermediate concentrations, with values between the lowest observed for AHH and the highest for AHP (Figure S6). Cu is an essential micronutrient in plants, involved in a wide range of physiological and biochemical processes. It plays a key role in chlorophyll synthesis, cell wall metabolism, oxidative stress responses, hormone signaling, transcription regulation, and protein trafficking. Additionally, Cu is required for oxidative phosphorylation, iron mobilization, and the biogenesis of the molybdenum cofactor, which is crucial for enzymatic reactions in nitrogen and sulfur metabolism [22]. Despite its importance, Cu levels must be tightly regulated, as both deficiencies and excesses can disrupt cellular homeostasis, leading to impaired growth and oxidative damage [23]. Pharmacologically, Cu contributes to antioxidant activity in plant extracts, as it is a cofactor for enzymes such as superoxide dismutase [24].

Other elements, such as Fe and Mn, displayed significant deviations between species. For instance, the concentrations of these elements differed by 1–3 orders of magnitude between AHH, AXC, and AHP (Figures S4 and S5). Iron is another vital element for plant metabolism, participating in fundamental processes such as photosynthesis, respiration, and nitrogen fixation. As a redox-active metal, Fe serves as a cofactor in numerous enzymatic reactions, facilitating electron transfer and ATP synthesis [25]. However, its reactivity also makes it potentially toxic at high concentrations, as it can generate reactive oxygen species (ROS) through Fenton reactions, leading to oxidative stress [22]. Similarly, Mn is an essential micronutrient that plays a fundamental role in plant growth and development [26,27]. It is particularly important in photosynthesis, where it is a key component of the oxygen-evolving complex, facilitating water splitting and oxygen production [28,29]. Mn also contributes to enzyme activation, protein metabolism, and resistance to biotic and abiotic stress. While Mn deficiencies can impair photosynthetic efficiency and overall plant health, excessive Mn accumulation can lead to toxicity, disrupting metabolic functions and competing with other essential metal ions [30,31].

Phosphorus (P), which was the second most abundant element after K, is shown in Figure S1. As a vital component of phospholipids, phosphoglucides, and phosphoproteins, P plays a crucial role in numerous biochemical processes. In the ash samples, P was likely present as phosphate ions, serving as a salting agent for metal cations. The P distribution followed a dynamic trend: AHP showed the lowest concentrations (approximately 30% lower than AXC), AHH occupied an intermediate position (approximately 10% lower than AXC), and AXC exhibited the highest levels. These results suggest that P concentration serves as a biomarker, supporting the hypothesis that AHH is a hybrid of AHP and AXC. However, in the absence of corroborating literature, genetic analysis is necessary to validate or refute this hypothesis. The elevated P levels in AXC may be attributed to more efficient phosphate uptake and storage mechanisms, possibly influenced by root physiology and symbiotic interactions with soil microorganisms. From a pharmacological perspective, P is essential for cellular metabolism, energy production, and bone health [32,33]. Higher P concentrations in AXC seeds may contribute to their nutritional value and potential therapeutic applications, particularly in energy-boosting and bone-strengthening herbal formulations.

(ii) Minor elements and potential microcontaminants.

The second group includes metals and metalloids present in lower concentrations, typically in the range of 10–1000 µg/100 g (d.b.). Elements in this group, ranked by maximum observed concentrations (µg/100 g, d.b.), included the following:

Si (983) > Pb (805) > Ni (696) > Mn (263) > Sr (84.5) > Se (31.8) > Cr (11.1).

Elements with concentrations below this range (1–10 µg/100 g, d.b.) are classified as a separate subset, with Cr at the borderline with an average concentration of 10.7 µg/100 g (d.b.) over four years of observation. However, the biological significance of many of these elements remains unclear, as their roles in plant metabolism are not well defined. Some of these elements may act as essential micronutrients, while others could be environmental contaminants with potential toxic effects. For instance, Ag and Ti were consistently detected in all samples, yet their function in plant systems remains uncertain. Some studies suggest that Ag can influence plant growth and stress responses [34], while Ti has been reported to enhance certain physiological processes, such as photosynthesis and nutrient uptake [35]. However, their persistent presence in plant tissues may also result from environmental contamination, emphasizing the need for further research on their bioavailability and potential accumulation in the food chain. Lithium serves as another example of an element naturally occurring in the environment but not classified as essential for biological systems [36]. Lithium toxicity in plants is known to disrupt physiological processes, alter metabolic pathways, and reduce growth. The mechanisms of Li uptake and transport in plants remain poorly understood, and its presence in seeds could indicate environmental exposure rather than a functional role in plant development [37]. Similar considerations apply to the other trace elements listed in Table 3. Although their concentrations are low and potential risks to plants, animals, and humans through consumption are minimal, their presence may reflect environmental factors such as soil composition, pollution sources, or anthropogenic influences. Lead (Pb) was detected at levels exceeding 800 µg/100 g, making it the second most abundant trace element in AXC seeds. Pb is a non-essential, toxic metal that can interfere with enzyme function, disrupt cellular processes, and cause oxidative damage in both plants and animals [38,39,40]. Although Pb uptake in plants is generally limited due to low mobility in soils, its high concentration in AXC seeds suggests a species-specific accumulation mechanism. The potential risks associated with Pb accumulation in AXC should be further evaluated, particularly in terms of its implications for medicinal applications. However, it is important to note that these values refer to the total metal content in the seeds and not to the composition of alcoholic extracts derived from them, which are the primary form used for pharmacological purposes. Similarly, Ni was detected at 696 µg/100 g, a level that is considerably higher than in many other plant species. Ni is essential in small amounts for plants, primarily as a cofactor in urease enzymes that facilitate nitrogen metabolism [41,42,43]. However, at elevated concentrations, Ni can cause toxicity symptoms such as chlorosis, stunted growth, and disruption of essential metal uptake (e.g., Fe and Zn). The high Ni levels in AXC warrant further investigation to determine whether its presence is linked to anthropogenic pollution or plant-specific absorption traits.

To provide a more comprehensive overview, Table 4 presents the elemental analysis data for soil samples collected at a depth of 20 cm near the root systems of the five *Aesculus* plants. The values in Table 4 represent the typical average composition of alluvial agronomic soils in Northern Italy. Furthermore, these results are consistent with the data obtained in a previous study conducted on AHP and AHH seeds [9].

The elemental composition of AXC seeds is influenced by both genetic factors and environmental conditions, particularly soil composition. The presence and concentration of elements in seeds depend on uptake efficiency, translocation mechanisms, and storage capacity within plant tissues. Comparing the elemental profiles of AXC seeds with the soil from which they were cultivated provides valuable insights into species-specific metal absorption patterns and potential bioaccumulation. A direct comparison between Table 3 and Table 4 reveals that several elements, including Fe, Cu, Zn, Mn, and P, are present in both soil and seeds, indicating their effective uptake and accumulation. Elements such as K, Ca, and Mg, essential for physiological functions, are found in significantly higher concentrations in seeds than in soil, suggesting active transport and preferential storage. Conversely, elements such as Ni and Pb, which are detected at relatively low concentrations in soil, are present in significantly higher amounts in AXC seeds, raising questions about species-specific metal accumulation mechanisms. The detection of trace elements such as Ag, Ti, and Li in seeds despite their minimal presence in soil suggests that AXC may possess unique absorption and retention capacities. In particular, the consistent presence of Li, a non-essential element known for its potential toxicity to plants, warrants further investigation into its uptake pathways and possible environmental exposure sources. Similarly, the presence of elements like Cr and Cd at measurable levels in seeds, despite their relatively low soil concentrations, highlights the need for additional studies on metal mobility, plant detoxification mechanisms, and potential implications for medicinal applications.

### 2.2. Analysis of Crude Extracts Obtained by Hydroalcoholic Maceration

#### Optimization of the Extraction Method

The preparation of materials intended for pharmacological applications or as food supplements with recognized therapeutic properties requires rigorous extraction and formulation methodologies. These methodologies are well established and validated through standardized procedures outlined in the current pharmacopeias [5].

To assess and compare the efficiency of different extraction techniques, key parameters, such as dry residue and ash content (indicative of the inorganic fraction), were evaluated. Table 5 summarizes the results of comparing the hydroalcoholic macerative extraction and sonication extraction methods for producing mother tinctures from AXC seed flours.

Sonication with 75% ethanol increased the extraction yield by approximately 30% compared to the 21-day maceration method. The comparative evaluation, based on the dry residue and ash content (representing the inorganic fraction), highlights the superior efficiency of sonication. The time advantage of sonication is exponential, reducing the extraction time from 21 days to 30 min.

Table 6 and Table 7 present the concentrations of selected elements in the hydroalcoholic extracts obtained via maceration and sonication of flour from the seeds of five AXC trees.

The observed trends in the elemental composition of the hydroalcoholic extracts are consistent with those reported for mineralized seed samples, allowing the classification of elements into two groups based on their concentration and extractability. However, significantly lower elemental concentrations were observed in the extracts compared to the raw seeds. This reduction is particularly advantageous in minimizing the presence of heavy metals, thereby improving the safety of the extracts. At the same time, it also results in a decreased retention of essential macroelements, such as K, P, and Ca, which may influence the nutritional contribution of the final preparation. The major elements, with concentrations ranging from 1 to 150 mg/100 g, included Ca, K, Mg, and P in extracts obtained via long-term maceration. In contrast, sonicated extracts additionally contained Fe and Na, suggesting that ultrasound-assisted extraction may enhance the solubilization of specific mineral components. The secondary elements, present at concentrations below 1000 µg/100 g include various trace components. Among these, As, B, Cd, Li, Mo, Sb, Ti, and V were either undetectable (n.d.) or completely absent in both extraction methods. This observation, consistent with the mineralized sample data (Table 3), suggests that the selected hydroalcoholic extraction methods do not effectively mobilize these metal cations or their hydroxylated metalloid species into the solution. Importantly, particularly toxic elements such as Cd, Pb, and Ni were detected at concentrations well below the limits established by the European Union for beverages produced by infusion or decoction [44]. This suggests that the extraction process significantly reduces the presence of these harmful metals, ensuring compliance with regulatory safety standards for human consumption.

For comparative purposes, Table 8 summarizes the average elemental composition of crude extracts (mother tinctures) prepared using both extraction methods over a four-year study period, along with the composition of a commercial herbal product (AXC_herb) produced via a 21-day hydroalcoholic maceration (based on the manufacturer’s label, anonymized). The commercial product analyzed in 2017 exhibited significantly lower (*p* < 0.05) concentrations of all analytes than our results.

Notably, K and P were present in relatively high concentrations in the commercial product (mg/100 mL scale), although the values were approximately 60–70% lower than those of our long-term macerates and over 70–80% lower than those obtained using sonication. Similarly, the ash and dry residue values, presented at the top of Table 8, indicate lower analyte concentrations in the commercial product than in the formulations prepared in this study. Given that such phytopharmacological products are heavily diluted prior to consumption for therapeutic or dietary purposes, the modest concentrations of active ingredients in commercial products raise questions about their efficacy in producing significant health effects. The hydroalcoholic composition of the solvent mixture significantly influences the extraction process. The weakly alkaline pH values of these solutions correlated with the concentration of salts (ash content) and the presence of alkalizing metals such as K, Mg, and Ca. In conclusion, the sonication method not only offers a substantial improvement in extraction efficiency and yield over traditional maceration but also provides a faster and more practical alternative. This method enhances the concentration of key active ingredients and ensures high-quality formulations for pharmacological and dietary applications.

## 3. Materials and Methods

### 3.1. Sample Collection

Five *Aesculus × Carnea* (AXC) trees located on the university campus of Modena (Italy) were selected as sources of plant material. The selected trees are approximately 70 years old and exhibit uniform morphological characteristics. Their average dimensions are as follows: a height of 8–9 m, a trunk diameter of 50–60 cm measured at 1 m above ground level, and a crown diameter of 8 m when projected onto the ground at solar noon. From each tree, 40–50 seeds were collected during the full maturation period, typically at the end of September or in the first ten days of October, when the spiny capsids reached the phenological opening phase. These same five trees provided seeds for four consecutive years (2016–2019). In 2019, soil samples were collected by coring the immediate vicinity of each selected tree. The samples were used to analyze soil composition and its potential influence on seed metal content.

Upon collection, the seeds were meticulously cleaned, weighed, and placed in a desiccator in the dark at room temperature for three months. This setup allowed natural drying under controlled conditions, preventing any alterations to the analyte matrix.

### 3.2. Sample Handling

For the analytical procedures, 10–15 seeds were randomly selected from each batch, and the total mass was reweighed to account for moisture loss during drying. The seeds were carefully dehulled, removing the woody pericarp and thin red-brown mantle sublayer (endosperm), which are closely associated with the hardened starchy-protein epicarp. Cotyledon fragments were collected, ground using an agate mortar to produce flour, and stored in polyethylene (PE) bags under vacuum to prevent oxidative degradation. The samples were then refrigerated at 0 °C until further analysis. Due to the nature of the seeds, substantial waste from the epicarp and episperm reduced the flour yield to approximately 25–30% of the fresh seed mass. This preparation procedure was consistently applied to all samples collected between 2016 and 2019.

### 3.3. Crude Extract Preparation

Each year, two different extraction procedures were applied to prepare crude extracts (mother tinctures [5]) using flour derived from seeds collected from the selected trees.

Hydroalcoholic maceration: To ensure optimal extraction efficiency, this method was performed in the dark at room temperature for 21 days. Several variables were optimized, including extraction method, solvent composition, temperature, and extraction time. Binary hydroalcoholic mixtures containing 25, 50, and 75% ethanol were tested as solvents. Preliminary experiments conducted in 2016 indicated that a binary mixture of 25% water and 75% ethanol provided superior extraction efficiency, both qualitatively and quantitatively, making it particularly well suited for intended applications.

Sonication extraction: This method involved mixing 10.0 ± 0.1 g of finely milled, undifferentiated seed flour with 100 mL of a binary solvent consisting of 96% ethanol. The mixture was then sonicated at 60 °C for 30 min. Following the sonication process, the material underwent chemical-physical treatment, which included filtration and washing of the solid residue. Washing was continued until the solvent volume was restored to its initial level. To optimize the extraction process, different extraction durations were tested (10, 20, 30, 45, and 60 min). Preliminary experiments revealed that the extraction yields plateaued beyond 30 min, identifying this as the optimal duration for the procedure. Sonication extraction was carried out using a Bransonic M1800-E (Emerson Electric Co., St. Louis, MO, USA) ultrasonic bath.

### 3.4. Mineralization Procedure

Prior to the operational procedure, all disposable materials and glassware were thoroughly cleaned by washing with a 10% HNO_3_ solution, followed by rinsing with deionized water (Milli-Q Plus, Millipore, Burlington, MA, USA). The six Teflon-coated vessels (model XP 1500+) used in a microwave carousel digestion system were preconditioned with 6 mL of HNO_3_ (65%) and 4 mL of H_2_O_2_ (30%), followed by a blank digestion cycle. This cleaning procedure was repeated after each mineralization round to ensure reproducibility.

Samples of floured AXC seeds (0.2–0.3 g) and of AXC extracts (1–2 mL) were digested in duplicate for each tree, along with one fortified sample to assess recovery factors, using the same ultrapure reagent mixture. Digestion was performed in an MW system (CEM MARSX; Charlotte, NC, USA), and the resulting solutions were diluted to 25 or 50 mL with ultrapure water, yielding clear, colorless, and stable solutions without turbidity. The MW digestion cycle conditions were as follows: 2 min at 250 W, 2 min at 0 W, 6 min at 250 W, 5 min at 400 W, and 8 min at 550 W, followed by an 8 min venting phase. These parameters were selected to optimize digestion efficiency in terms of time and recovery for these natural matrices. Soil samples were analyzed following the method reported in a previous study [45].

Table 9 provides a summary of the sample numbers and preparatory treatments used for subsequent ICP OES analysis.

### 3.5. Reagents and Standard

All mineral acids and oxidants (HNO_3_ 65% and H_2_O_2_ 30%) were of the highest purity grade (Suprapur^®^) and were obtained from Merck (Darmstadt, Germany). An ICP single-element standard (As, B, Na, K) and multi-element standards containing 22 elements (Ag, Al, Ca, Cd, Co, Cr, Cu, Fe, Li, Mg, Mn, Mo, Ni, P, Pb, Sb, Se, Si, Sr, Ti, V, Zn) were obtained from Merck. They were used to prepare reference solutions for calibration curves, as well as fortified and spiked samples, and were used at concentrations ranging from 10 to 1000 mg/L. Ethanol (96%), acetonitrile, and diethyl ether were obtained from Carlo Erba Reagents (Milan, Italy). Deionized water was produced using a Milli-Q Plus system (Millipore, Merck, Darmstadt, Germany).

### 3.6. Proximate Analysis

Each measurement was repeated at least three times, and the final data were expressed on a dry matter basis, determined by the total desiccation of fresh fruit in an oven at 105 °C for 4 h until a constant weight was achieved. Quantitative data on the residual moisture in the seeds after natural drying for 3 months were also obtained.

Total protein was quantified using the Kjeldahl method with a CuSO_4_-based catalyst [46]. A Gerhardt Kjeldatherm system equipped with a distillation unit (Gerhardt Vapodest, Gerhardt GmbH & Co., Königswinter, Germany) was used for digestion. The nitrogen percentage was converted into protein mass content by multiplying by a conversion factor of 4.86, which is specific to chestnuts and assumed to be appropriate for horse chestnuts as well [47].

The crude fat content was quantified using the AOAC method [46] by extracting the samples with diethyl ether for 3 h in a Soxhlet apparatus. After evaporation, the residue was dried for 2 h at 105 °C until a constant weight was obtained.

The total sugar content in the dried floured samples was determined by hydroalcoholic extraction (80/20 *v*/*v*, water/ethanol) using a sonicator with a thermostat bat at 60 °C for 30 min. After microfiltration through a 2 μm PE membrane, the extract was injected into an HPLC system. The instrumental setup and experimental conditions were described in our previous work [6].

The total mineral content was determined by incinerating and calcining the original matrix following the AOAC method [46].

Cold water solubility (CWS%) was determined at 100 °C using a slightly modified method adapted for these complex matrices. The detailed experimental procedure was described in our previous article [6].

Total inorganic soluble salt (TISS) content was measured starting from the dried material obtained from CWS by incineration and calcination at 550 °C until a constant weight was achieved.

CHNS elemental analysis was conducted with a CHNS Analyzer Flash2000 (Thermo-Scientific, Waltham, MA, USA).

### 3.7. ICP OES Analysis

The metal content was determined using ICP-OES with a Perkin-Elmer Optima 4200 DV spectrometer equipped with an ultrasonic nebulizer (Cetac Technologies Inc., Omaha, NE, USA). A Charge-Coupled Device (CCD) area detector was used to determine the total elemental content. The floured seed samples (0.2–0.3 g) were mineralized using a mixture of HNO_3_ 65% (6 mL) and H_2_O_2_ 30% (4 mL) in a CEM MARSX microwave system (Charlotte, NC, USA), equipped with Teflon-coated carousel vessels (model XP 1500+). The samples were then diluted to a final volume of 25 mL or 50 mL, depending on the need, using deionized water from a Milli-Q system. All experimental details and instrumental setup have been previously described [9]. Additional details on the emission wavelengths (λ), Limit Of Quantification (LOQ) and Linear Operative Limit (LOL) values, linear correlation coefficient (*r*), and relative standard deviation (RSD) of the external calibration and recovery factor for each analyzed element are provided in Table S1.

### 3.8. Data Analysis

The results are expressed as means ± standard deviations of the mean and statistically analyzed using Matlab 2023a software. For comparison results, a one-way analysis of variance (ANOVA) test followed by a Tukey–Kramer post hoc ANOVA test was used, and *p* values of less than 0.05 were considered statistically significant.

## 4. Conclusions

This study characterized the metallic fraction of *Aesculus × carnea* (AXC) seeds over four consecutive years (2016–2019), using material collected from Modena (Italy). Compared to AHP and AHH, AXC seeds exhibited significantly higher metal concentrations—approximately 20–30% higher across most analyzed elements.

Hydroalcoholic extracts (mother tinctures) were obtained using two methods: maceration (21 days, room temperature) and sonication (30 min, 60 °C).

Sonication significantly improved the extraction yield (by approximately 30%) while reducing the processing time. Comparison with a commercial herbal product revealed that AXC extracts contained approximately 30% higher concentrations of the measured analytes.

Given the complexity of the matrix and the pharmacological relevance of its components, further studies are needed to elucidate the fate of metal cations and bioactive compounds in both solid and solution phases. These investigations will contribute to a deeper understanding of the chemical nature and therapeutic potential of AXC and its related plant matrices.

## Figures and Tables

**Table 1:** Biometric parameters and physical characteristics of the samples of red horse-chestnut seeds from AXC trees, harvested in different years (2016–2019).

	2016	2017	2018	2019	Mean
Mass * (g) (min-max)	10.7 (7.46–21.2)	9.96 (8.07–18.5)	10.3 (8.16–22.3)	10.1 (7.94–19.5)	10.3 (7.46–22.3)
No. of seeds/kg	93.1	100	97.0	98.5	97.2
Bark-crust%	18.9	20.6	19.5	19.8	19.7
Kernel%	81.1	79.4	80.5	80.2	80.3
Shape	Typical, almost irregular, ovoid, or more rounded and flattened.				

* mean values related to at least 100 seeds from 5 trees.

**Table 2:** Chemical composition of the red horse-chestnut seeds (AXC genotype). Data are expressed as mean ± standard deviation of 3 replicates and are expressed on a dry basis. Differences between means indicated by the same letters are not statistically significant (*p* < 0.05).

	2016	2017	2018	2019
Humidity% *	50.1 ± 0.9 ^a^	50.6 ± 0.7 ^a^	50.8 ± 0.6 ^a^	50.3 ± 0.7 ^a^
Moisture% ^#^	10.5 ± 0.4 ^a^	10.4 ± 0.3 ^a^	10.1 ± 0.4 ^a^	10.0 ± 0.5 ^a^
Proteins%	3.25 ± 0.44 ^a^	3.16 ± 0.40 ^a^	3.11 ± 0.37 ^a^	3.37 ± 0.42 ^a^
Lipids%	4.56 ± 0.48 ^a^	4.34 ± 0.42 ^a^	4.41 ± 0.38 ^a^	4.27 ± 0.46 ^a^
Carbohydrates%	15.8 ± 0.7 ^a^	15.6 ± 0.7 ^a^	15.2 ± 0.6 ^a^	15.3 ± 0.6 ^a^
Ashes%	3.08 ± 0.10 ^a^	3.14 ± 0.10 ^a^	3.20 ± 0.11 ^a^	3.10 ± 0.13 ^a^
CWS%	54.9 ± 0.4 ^a^	55.1 ± 0.6 ^a^	54.8 ± 0.5 ^a^	54.5 ± 0.6 ^a^
TISS%	2.69 ± 0.18 ^a^	2.79 ± 0.15 ^a^	2.81 ± 0.12 ^a^	2.73 ± 0.16 ^a^
C%	44.63 ± 0.19 ^a^	44.92 ± 0.23 ^a^	44.59 ± 0.21 ^a^	44.10 ± 0.27 ^a^
H%	6.34 ± 0.14 ^a^	6.25 ± 0.14 ^a^	6.12 ± 0.17 ^a^	6.58 ± 0.15 ^a^
N%	1.49 ± 0.12 ^a^	1.52 ± 0.10 ^a^	1.32 ± 0.13 ^a^	1.41 ± 0.11 ^a^
S%	<0.1	<0.1	<0.1	<0.1

* determined on the fresh seeds, ^#^ residual moisture after natural drying.

**Table 3:** Element concentrations in red horse-chestnut seeds flour from AXC, harvested in different years. Differences between means indicated by the same letters are not statistically significant (*p* < 0.05).

	2016	2017	2018	2019
	Major elements (mg/100 g)			
Al	3.10 ± 0.30 ^a^	3.27 ± 0.42 ^a^	3.12 ± 0.34 ^a^	3.19 ± 0.24 ^a^
Ca	51.5 ± 2.1 ^a^	57.3 ± 4.6 ^ab^	59.9 ± 1.3 ^b^	54.2 ± 1.9 ^ab^
Cu	1.77 ± 0.15 ^a^	1.81 ± 0.14 ^a^	1.95 ± 0.12 ^a^	1.82 ± 0.13 ^a^
Fe	4.59 ± 0.48 ^a^	4.65 ± 0.42 ^a^	4.51 ± 0.34 ^a^	4.30 ± 0.38 ^a^
K	1322 ± 103 ^a^	1392 ± 136 ^a^	1398 ± 78 ^a^	1476 ± 88 ^a^
Mg	91.5 ± 6.8 ^a^	96.3 ± 9.3 ^a^	100.4 ± 6.6 ^a^	96.8 ± 4.1 ^a^
Na	9.39 ± 0.43 ^a^	9.35 ± 0.61 ^a^	9.08 ± 0.55 ^a^	9.23 ± 0.56 ^a^
P	858 ± 50 ^a^	869 ± 78 ^a^	871 ± 58 ^a^	854 ± 46 ^a^
Zn	1.75 ± 0.25 ^a^	1.82 ± 0.20 ^a^	1.78 ± 0.18 ^a^	1.90 ± 0.18
	Minor elements (μg/100 g)			
Ag	2.18 ± 0.26 ^a^	2.27 ± 0.31 ^a^	2.41 ± 0.17 ^a^	2.03 ± 0.14 ^a^
As	3.55 ± 0.29 ^a^	3.71 ± 0.26 ^a^	3.18 ± 0.11 ^a^	3.61 ± 0.20 ^a^
B	1.61 ± 0.13 ^ab^	1.57 ± 0.21 ^ab^	1.52 ± 0.17 ^b^	1.74 ± 0.10 ^a^
Cd	2.09 ± 0.34 ^a^	1.97 ± 0.56 ^a^	2.01 ± 0.53 ^a^	2.05 ± 0.20 ^a^
Co	4.36 ± 0.43 ^a^	4.57 ± 0.82 ^a^	4.82 ± 0.54 ^a^	3.91 ± 0.51 ^a^
Cr	10.8 ± 1.0 ^a^	11.0 ± 2.3 ^a^	10.2 ± 1.1 ^a^	11.1 ± 0.6 ^a^
Li	1.74 ± 0.29 ^a^	1.68 ± 0.16 ^b^	1.53 ± 0.29 ^ab^	1.61 ± 0.22 ^ab^
Mn	256 ± 57 ^a^	249 ± 46 ^a^	263 ± 49 ^a^	238 ± 53 ^a^
Mo	1.20 ± 0.16 ^a^	1.98 ± 0.18 ^b^	1.68 ± 0.10 ^b^	1.51 ± 0.11 ^a^
Ni	606 ± 86 ^a^	647 ± 42 ^a^	635 ± 47 ^a^	693 ± 48 ^a^
Pb	742 ± 53 ^a^	771 ± 79 ^a^	805± 54 ^a^	713 ± 42 ^a^
Sb	2.62 ± 0.34 ^ab^	2.52 ± 0.17 ^ab^	2.95 ± 0.27 ^a^	2.17 ± 0.26 ^b^
Se	254 ± 33 ^a^	287 ± 39 ^a^	318 ± 60 ^a^	307 ± 49 ^a^
Si	892 ± 52 ^a^	983 ± 44 ^a^	952 ± 68 ^a^	918 ± 66 ^a^
Sr	82.2 ± 4.3 ^a^	83.2 ± 5.5 ^a^	78.2 ± 4.9 ^a^	84.5 ± 6.0 ^a^
Ti	1.72 ± 0.08 ^a^	1.32 ± 0.13 ^b^	1.70 ± 0.08 ^a^	1.60 ± 0.07 ^ab^
V	2.46 ± 0.17 ^a^	2.73 ± 0.18 ^a^	2.31 ± 0.19 ^ab^	2.10 ± 0.20 ^b^

**Table 4:** Concentrations of some elements (mg/100 g on a dry basis) in top-soil samples, representative of the growing trees of the AXC seed samples. Data collected in 2019. Data are expressed as mean ± standard deviation of three replicated.

Element	Concentration (mg/100 g)
Ag	0.0004 ± 0.0003
Al	5739 ± 178
As	0.84 ± 0.10
B	2.27 ± 0.17
Bi	1.95 ± 0.13
Ca	3934 ± 262
Cd	0.23 ± 0.03
Co	2.66 ± 0.16
Cr	9.67 ± 0.19
Cu	10.8 ± 0.6
Fe	3078 ± 262
Ga	2.82 ± 0.12
In	1.31 ± 0.17
K	2998 ± 197
Li	1.05 ± 0.11
Mn	454.7 ± 38.0
Mg	121.1 ± 5.6
Mo	1.08 ± 0.08
Na	2866 ± 121
Ni	5.71 ± 0.32
P	55.4 ± 2.2
Pb	10.7 ± 0.5
Sb	0.103 ± 0.009
Se	0.041 ± 0.005
Si (%)	20.7 ± 0.2
Sr	30.9 ± 1.8
Ti	1.64 ± 0.09
Tl	0.035 ± 0.014
V	3.37 ± 0.20
Zn	14.3 ± 1.1

**Table 5:** Evaluation parameters of extraction yield for the production of mother tincture from AXC seed flour (2016–2019).

	2016	2017	2018	2019
	Hydroalcoholic macerative extraction			
Dry Residue (g/100 mL)	1.146 ± 0.118	1.262 ± 0.103	1.175 ± 0.115	1.233 ± 0.092
Ashes (mg/100 mL)	125.6 ± 4.8	143.9 ± 3.9	137.7 ± 4.3	144.8 ± 3.7
	Hydroalcoholic extraction by sonication			
Dry Residue (g/100 mL)	1.215 ± 0.122	1.279 ± 0.141	1.288 ± 0.136	1.287 ± 0.105
Ashes (mg/100 mL)	173.3 ± 5.2	204.2 ± 6.1	195.5 ± 5.5	181.7 ± 4.6

**Table 6:** Concentrations of some elements in crude extracts of AXC flour (mother tincture), obtained through maceration for 21 days in the dark at room temperature. Differences between means indicated by the same letters are not statistically significant (*p* < 0.05).

	2016	2017	2018	2019
	Major elements (mg/100 g)			
Ca	1.39 ± 0.11 ^a^	1.17 ± 0.11 ^a^	1.49 ± 0.18 ^ab^	1.45 ± 0.11 ^b^
K	97.9 ± 12.1 ^a^	90.9 ± 5.8 ^a^	95.0 ± 8.6 ^a^	93.7 ± 8.7 ^a^
Mg	3.90 ± 0.14 ^a^	4.01 ± 0.08 ^a^	3.41 ± 0.19 ^b^	3.69 ± 0.12 ^ab^
P	16.7 ± 2.5 ^a^	16.1 ± 3.2 ^a^	18.2 ± 1.9 ^a^	15.1 ± 1.8 ^a^
	Minor elements (μg/100 g)			
Ag	0.15 ± 0.03 ^ab^	0.09 ± 0.03 ^a^	0.12 ± 0.02 ^ab^	0.17 ± 0.03 ^b^
Al	232 ± 54 ^a^	263 ± 44 ^a^	229 ± 39 ^a^	242 ± 76 ^a^
As	n.d.	n.d.	n.d.	n.d.
B	1.26 ± 0.14 ^a^	1.49 ± 0.14 ^a^	1.27 ± 0.19 ^a^	1.32 ± 0.14 ^a^
Cd	0.127 ± 0.035 ^a^	0.147 ± 0.035 ^a^	0.103 ± 0.021 ^a^	0.153 ± 0.040 ^a^
Co	1.37 ± 0.08 ^a^	1.69 ± 0.09 ^a^	1.23 ± 0.07 ^a^	1.41 ± 0.09 ^a^
Cr	1.29 ± 0.13 ^ab^	1.56 ± 0.14 ^a^	1.14 ± 0.10 ^b^	1.43 ± 0.12 ^ab^
Cu	180 ± 14 ^a^	158 ± 16 ^ab^	134 ± 18 ^b^	166 ± 18 ^ab^
Fe	789 ± 105 ^a^	749 ± 60 ^a^	628 ± 81 ^a^	795 ± 112 ^a^
Li	n.d.	n.d.	n.d.	n.d.
Mn	59.2 ± 9.4 ^a^	51.5 ± 7.6 ^a^	61.0 ± 7.8 ^a^	56.0 ± 8.2 ^a^
Mo	n.d.	n.d.	n.d.	n.d.
Na	767 ± 26 ^a^	968 ± 75 ^b^	939 ± 40 ^ab^	856 ± 58 ^b^
Ni	179 ± 12 ^ab^	196 ± 9 ^a^	158 ± 12 ^b^	165 ± 10 ^b^
Pb	8.07 ± 0.86 ^a^	8.98 ± 0.66 ^a^	7.84 ± 0.27 ^a^	7.69 ± 0.54 ^a^
Sb	n.d.	n.d.	n.d.	n.d.
Se	11.7 ± 3.0 ^a^	14.9 ± 3.6 ^a^	19.3 ± 4.2 ^a^	15.3 ± 4.3 ^a^
Si	29.9 ± 4.5 ^a^	38.8 ± 3.3 ^a^	31.5 ± 3.1 ^a^	39.7 ± 4.1 ^a^
Sr	8.23 ± 0.85 ^a^	7.73 ± 1.1 ^a^	9.64 ± 0.98 ^a^	11.6 ± 2.7 ^a^
Ti	n.d.	n.d.	n.d.	n.d.
V	n.d.	n.d.	n.d.	n.d.
Zn	126 ± 15 ^a^	102 ± 12 ^a^	117 ± 10 ^a^	122 ± 11 ^a^

n.d.: not detected.

**Table 7:** Concentrations of some elements in crude extracts of AXC flour (mother tincture), obtained through sonication for 30 min at 60 °C. Differences between means indicated by the same letters are not statistically significant (*p* < 0.05).

	2016	2017	2018	2019
	Major elements (mg/100 g)			
Ca	2.92 ± 0.26 ^a^	2.51 ± 0.18 ^a^	2.73 ± 0.12 ^a^	2.87 ± 0.11 ^a^
Fe	1.40 ± 0.20 ^a^	1.46 ± 0.22 ^a^	1.17 ± 0.12 ^a^	1.10 ± 0.14 ^a^
K	106 ± 12 ^a^	133 ± 14 ^a^	126 ± 16 ^a^	118 ± 18 ^a^
Mg	5.05 ± 0.19 ^a^	5.45 ± 0.18 ^a^	5.76 ± 0.19 ^a^	5.26 ± 0.17 ^a^
Na	1.46 ± 0.11 ^a^	1.22 ± 0.15 ^a^	1.53 ± 0.19 ^a^	1.41 ± 0.18 ^a^
P	19.4 ± 0.9 ^a^	15.7 ± 0.7 ^a^	19.0 ± 1.2 ^a^	17.6 ± 1.6 ^a^
	Minor elements (μg/100 g)			
Ag	0.51 ± 0.12 ^a^	0.73 ± 0.09 ^a^	0.65 ± 0.11 ^a^	0.59 ± 0.12 ^a^
Al	329 ± 40 ^a^	285 ± 51 ^a^	312 ± 26 ^a^	394 ± 42 ^a^
As	n.d.	n.d.	n.d.	n.d.
B	1.30 ± 0.16 ^a^	1.18 ± 0.17 ^a^	1.22 ± 0.14 ^a^	1.42 ± 0.16 ^a^
Cd	0.28 ± 0.08 ^a^	0.19 ± 0.07 ^a^	0.24 ± 0.07 ^a^	0.21 ± 0.07 ^a^
Co	1.69 ± 0.14 ^a^	1.27 ± 0.09 ^a^	1.56 ± 0.11 ^a^	1.85 ± 0.12 ^a^
Cr	1.79 ± 0.20 ^a^	1.51 ± 0.24 ^a^	1.23 ± 0.19 ^a^	1.98 ± 0.17 ^a^
Cu	297 ± 10 ^a^	227 ± 18 ^a^	266 ± 14 ^a^	243 ± 15 ^a^
Li	n.d.	n.d.	n.d.	n.d.
Mn	0.61 ± 0.10 ^a^	227 ± 18 ^a^	266 ± 14 ^a^	243 ± 15 ^a^
Mo	n.d.	n.d.	n.d.	n.d.
Ni	234 ± 22 ^a^	276 ± 17 ^a^	215 ± 15 ^a^	228 ± 16 ^a^
Pb	9.58 ± 0.32 ^a^	9.10 ± 0.23 ^a^	9.24 ± 0.12 ^a^	9.82 ± 0.12 ^a^
Sb	n.d.	n.d.	n.d.	n.d.
Se	10.5 ± 2.5 ^a^	11.8 ± 2.7 ^a^	13.4 ± 3.0 ^a^	11.1 ± 3.1 ^a^
Si	51.6 ± 5.1 ^a^	61.1 ± 6.0 ^a^	57.8 ± 9.6 ^a^	68.3 ± 7.8 ^a^
Sr	17.0 ± 5.1 ^a^	21.0 ± 5.8 ^a^	18.5 ± 6.1 ^a^	17.5 ± 5.4 ^a^
Ti	n.d.	n.d.	n.d.	n.d.
V	n.d.	n.d.	n.d.	n.d.
Zn	152 ± 33 ^a^	137 ± 22 ^a^	158 ± 45 ^a^	184 ± 25 ^a^

n.d.: not detected.

**Table 8:** Average elemental composition of crude extracts (calculated from Table 7 and Table 8) compared to a commercial herboristic product. Differences between means indicated by the same letters are not statistically significant (*p* < 0.05).

	Maceration	Sonication	AXC_Herb
Ethanol%	75%	75%	38%
pH	7.5 ± 0.1	7.6 ± 0.2	7.4 ± 0.1
Dry residue (g/100 mL)	1.204 ± 0.108	1.269 ± 0.131	1.05 ± 0.06
Ashes (mg/100 mL)	138.0 ± 4.22	188.7 ± 5.3	40.8 ± 0.3
Element	mg/100 mL		
K	94.6 ± 9.1 ^a^	121 ± 15 ^a^	34.4 ± 7.8 ^b^
P	16.1 ± 2.1 ^a^	17.9 ± 0.9 ^a^	3.58 ± 0.42 ^b^
	μg/100 mL		
Ag	0.14 ± 0.02 ^a^	0.75 ± 0.11 ^b^	n.d.
Al	249 ± 56 ^a^	316 ± 41 ^b^	149 ± 16 ^c^
As	n.d.	n.d.	n.d.
B	1.33 ± 0.08 ^a^	1.28 ± 0.16 ^a^	n.d.
Ca	1382 ± 131 ^a^	2763 ± 178 ^b^	432 ± 27 ^c^
Cd	0.13 ± 0.01 ^a^	0.23 ± 0.04 ^b^	n.d.
Co	1.38 ± 0.08 ^a^	1.57 ± 0.12 ^b^	n.d.
Cr	1.36 ± 0.12 ^a^	1.67 ± 0.20 ^a^	n.d.
Cu	159 ± 17 ^a^	258 ± 15 ^b^	55.5 ± 6.1 ^c^
Fe	741 ± 92 ^a^	1280 ± 175 ^b^	110 ± 11 ^c^
Li	n.d.	n.d.	n.d.
Mg	3752 ± 138 ^a^	5384 ± 182 ^b^	89.7 ± 6.0 ^c^
Mn	57.7 ± 8.3 ^a^	72.5 ± 7.5 ^a^	25.5 ± 5.3 ^b^
Mo	n.d.	n.d.	n.d.
Na	882 ± 53 ^a^	1413 ± 161 ^b^	772 ± 115 ^a^
Ni	180 ± 11 ^a^	236 ± 18 ^b^	66.6 ± 4.5 ^c^
Pb	7.95 ± 0.62 ^a^	9.39 ± 0.21 ^b^	5.93 ± 0.09 ^c^
Sb	n.d.	n.d.	n.d.
Se	14.1 ± 3.8 ^a^	11.7 ± 2.8 ^b^	0.26 ± 0.03 ^c^
Si	33.7 ± 3.8 ^a^	59.9 ± 6.5 ^b^	12.2 ± 0.4 ^c^
Sr	9.35 ± 1.60 ^a^	18.5 ± 5.6 ^b^	n.d.
Ti	n.d.	n.d.	n.d.
V	n.d.	n.d.	n.d.
Zn	119 ± 13 ^a^	158 ± 32 ^b^	38.5 ± 3.4 ^c^

n.d.: not detected.

**Table 9:** Workplan for independent samples and methodologies (*) (#) for ICP OES determination across AXC seed samples (2016–2019) and soil samples (2019).

	2016–2019	Soil Samples (2019)
No. of trees	5	5
Replicates per tree	2 + 1 spiked * + 1 fortified ^#^	1 + 1 spiked * + 1 fortified ^#^
Total no. of replicates for five trees	10 + 5 spiked * + 5 fortified ^#^	5 + 5 spiked * + 5 fortified ^#^
Crude extracts from flour of AXC mixed seeds from five trees	2 + 1 spiked *	

* spiked with a multistandard (22 elements) after mineralization, ^#^ fortified with a multistandard (22 elements) before mineralization.

## Data Availability

Data are contained within the article.

## Supplementary Materials

The following supporting information can be downloaded at https://www.mdpi.com/article/10.3390/molecules30040819/s1, Table S1: Emission wavelengths (λ), Limit Of Quantification (LOQ) and Linear Operative Limit (LOL), linear correlation coefficient (*r*), and relative standard deviation (RSD) of the external calibration and recovery factor for each element determined; Figure S1: Comparison of AHP, AHH, and AXC samples in P concentrations; Figure S2: Comparison of AHP, AHH, and AXC samples in K concentrations; Figure S3: Comparison of AHP, AHH, and AXC samples in Zn concentrations; Figure S4: Comparison of AHP, AHH, and AXC samples in Mn concentrations; Figure S5: Comparison of AHP, AHH, and AXC samples in Fe concentrations; Figure S6: Comparison of AHP, AHH, and AXC samples in Cu concentrations.
