# Supplementary material for: Mineral Composition Analysis of Red Horse-Chestnut (Aesculus × Carnea) Seeds and Hydroalcoholic Crude Extract Using ICP OES
Source: Molecules. 2025 Feb 10;30(4):819. doi: 10.3390/molecules30040819 (PMC11858534; doi:10.3390/molecules30040819)
Supplement: Supplementary file 1 [file molecules-30-00819-s001.zip › molecules-3423974-supplementary.pdf]

Table S1 presents the emission wavelengths (λ), the selected Limit of Quantification (LOQ) and Linear Operative Limit, Linear Correlation Coefficient (r), relative standard deviation (RSD) of the external calibration and recovery factor for each element determined. The Limit of Quantification was defined as 3.3 times the LOD, which was defined as 3 times the standard deviation of ten measurements of the blank divided by the slope of the calibration curve.

Table S1. Emission wavelengths (λ), selected Limit Of Quantification (LOQ) and Linear Operative Limit (LOL), linear correlation coefficient (*r*), relative standard deviation (RSD) of the external calibration and recovery factor for each element determined.

Element	λ (nm)	LOQ ÷ LOL (mg L^{-1})	r	RSD%	Recovery%
Ag	328.068	0.005 ÷ 50	0.9995	4.9	96
Al	308.215<br>394.401	0.005 ÷ 20	0.9997	5.6	105
As	188.980<br>193.696	0.01 ÷ 50	0.9982	6.3	106
B	249.678<br>249.772	0.015 ÷ 50	0.9975	6.2	97
Bi	223.061<br>306.766	0.01 ÷ 10	0.9968	8.1	96
Ca	317.933<br>396.847	0.01 ÷ 100	0.9998	5.4	100
Cd	214.440<br>226.502	0.005 ÷ 10	0.9990	4.9	93
Co	228.616	0.005 ÷ 10	0.9979	3.5	94
Cr	267.716<br>283.563	0.005 ÷ 20	0.9978	11.3	98
Cu	324.752<br>327.393	0.005 ÷ 30	0.9999	5.0	99
Fe	238.204<br>259.939	0.005 ÷ 100	0.9997	4.5	97
Ga	294.364	0.03 ÷ 10	0.9983	3.7	98
In	325.609	0.03 ÷ 10	0.9990	2.8	99
K	766.490	0.01 ÷ 200	0.9995	7.2	94
Li	610.365<br>670.783	0.001 ÷ 50	0.9996	3.1	103

Mg	280.271<br>285.213	0.01 ÷ 200	0.9997	6.2	95
Mn	257.610<br>259.372	0.005 ÷ 40	0.9999	5.4	100
Mo	202.031<br>203.845	0.005 ÷ 30	0.9987	1.8	106
Na	589.592<br>330.237	0.01 ÷ 100	0.9998	4.6	95
Ni	231.604<br>341.476	0.005 ÷ 50	0.9999	6.7	102
P	213.617<br>214.914	10 ÷ 2000	0.9989	9.2	95
Pb	220.353<br>261.458	0.03 ÷ 40	0.9986	8.2	94
Sb	206.838<br>217.582	0.005 ÷ 30	0.9982	3.5	98
Se	196.026<br>203.985	0.01 ÷ 50	0.9998	5.1	96
Si	251.612<br>288.158	0.1 ÷ 10	0.9992	8.3	94
Sr	407.771<br>421.552	0.01 ÷ 50	0.9996	7.0	95
Ti	334.940<br>336.121	0.005 ÷ 100	0.9979	4.2	94
Tl	351.923	0.05 ÷ 50	0.9983	3.9	94
V	309.310	0.005 ÷ 40	0.9988	5.0	98
Zn	202.548<br>213.857	0.05 ÷ 50	0.9996	3.1	99

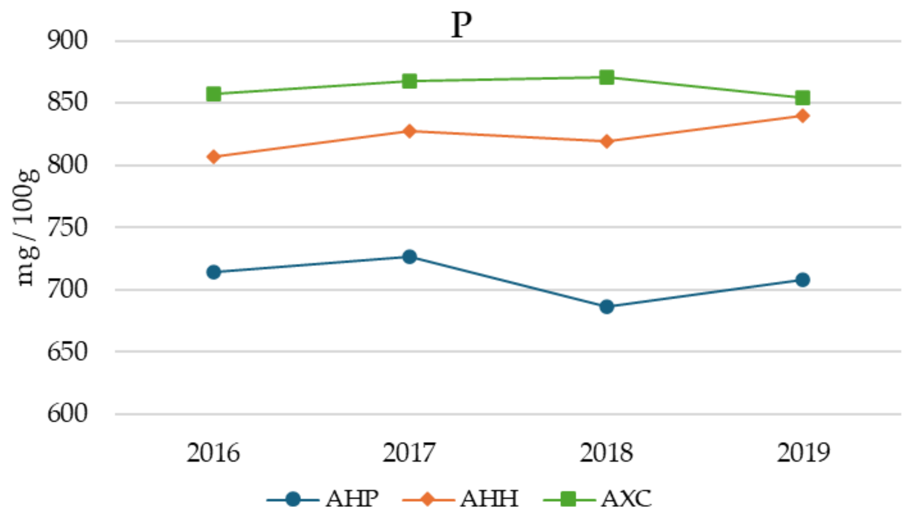

Figure S1. Comparison of AHP, AHH, and AXC samples in P concentrations.

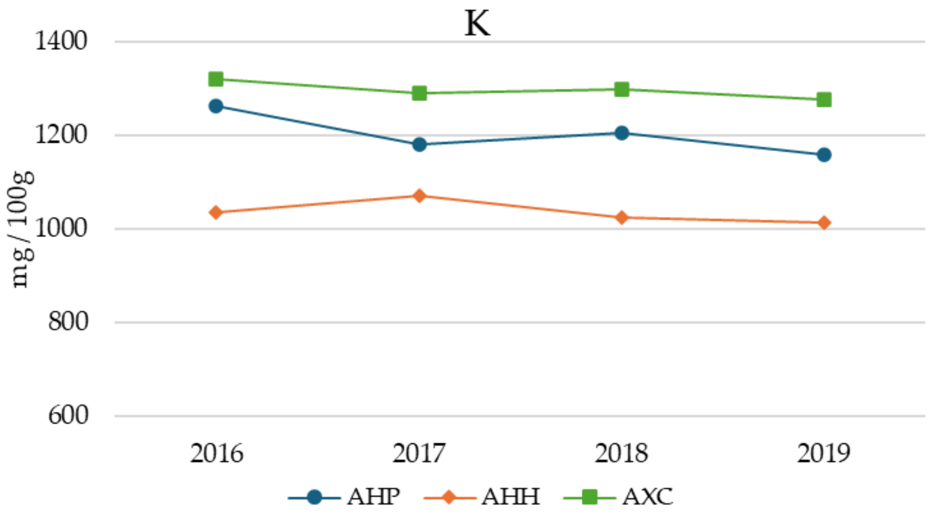

Figure S2. Comparison of AHP, AHH, and AXC samples in K concentrations.

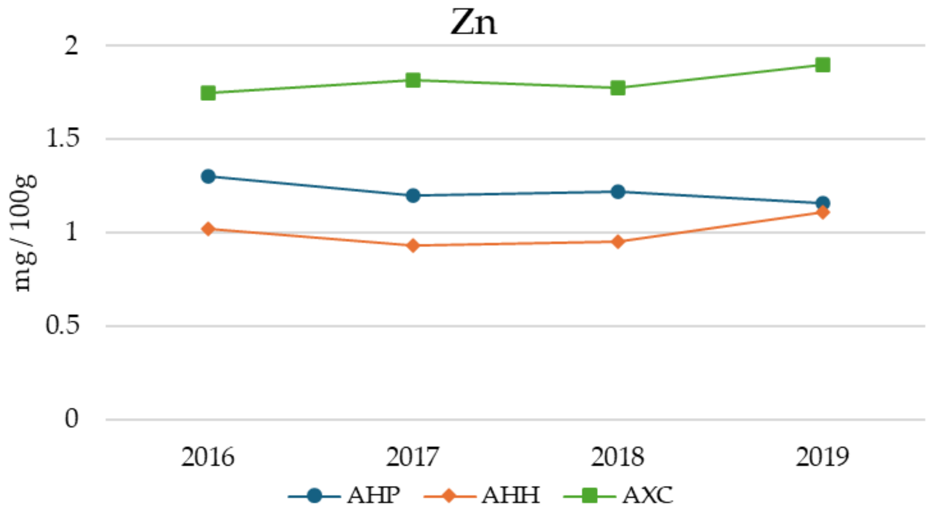

Figure S3. Comparison of AHP, AHH, and AXC samples in Zn concentrations.

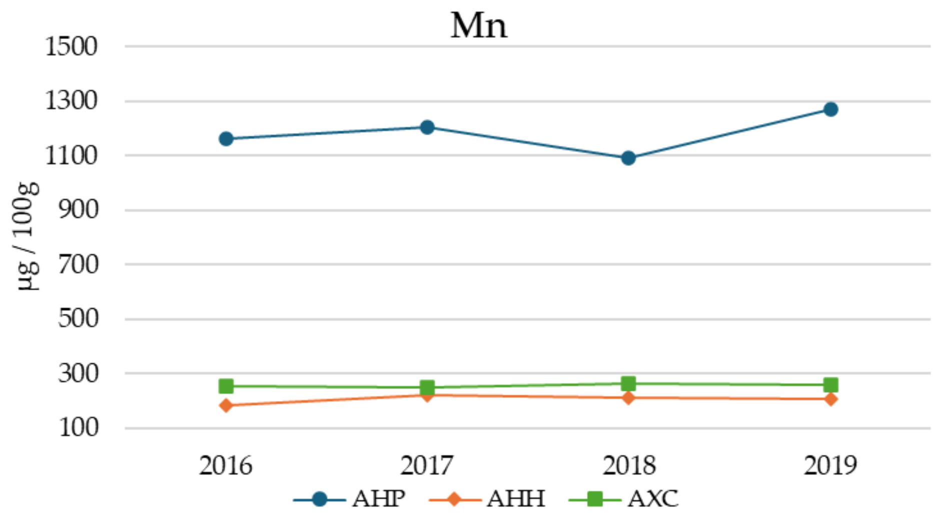

Figure S4. Comparison of AHP, AHH, and AXC samples in Mn concentrations.

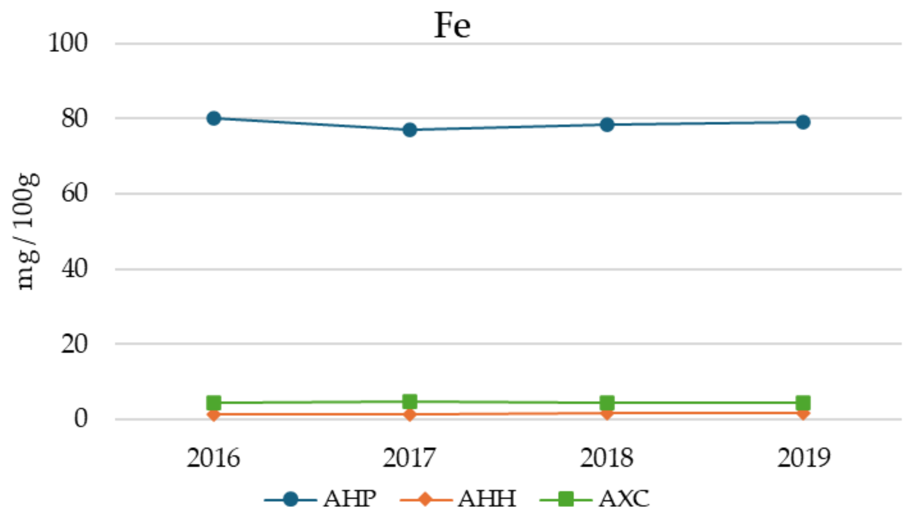

Figure S5. Comparison of AHP, AHH, and AXC samples in Fe concentrations.

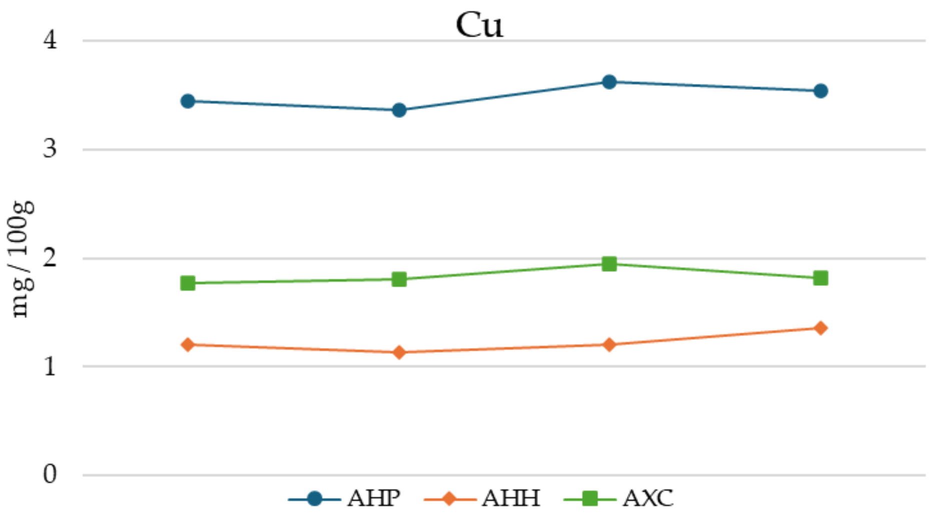

Figure S6. Comparison of AHP, AHH, and AXC samples in Cu concentrations.
